# A Case of Adult-Onset Still’s Disease Caused by a Novel Splicing Mutation in *TNFAIP3* Successfully Treated With Tocilizumab

**DOI:** 10.3389/fimmu.2018.01527

**Published:** 2018-07-04

**Authors:** Dylan Lawless, Shelly Pathak, Thomas Edward Scambler, Lylia Ouboussad, Rashida Anwar, Sinisa Savic

**Affiliations:** ^1^Leeds Institute of Biomedical and Clinical Sciences, University of Leeds, Wellcome Trust Brenner Building, St James’s University Hospital, Leeds, United Kingdom; ^2^Leeds Institute of Rheumatic and Musculoskeletal Medicine, Wellcome Trust Brenner Building, St. James’s University Hospital, Leeds, United Kingdom; ^3^Department of Clinical Immunology and Allergy, St. James’s University Hospital, Leeds, United Kingdom

**Keywords:** TNFAIP3, A20, AOSD, tocilizumab, autoinflammatory

## Abstract

*TNFAIP3* encodes the NF-κB regulatory protein A20. High-penetrance heterozygous mutations in *TNFAIP3* cause a haploinsufficiency of A20 (HA20), inadequate inhibition of NF-κB pathway, and an early onset autoinflammatory disorder. However, the clinical phenotype of patients with HA20 varies greatly and clinical diagnoses prior to establishing the genetic cause, included both autoimmune and autoinflammatory conditions. Here, we present the first patient with HA20, who was previously diagnosed with AOSD but was later found to have a novel heterozygous variant in *TNFAIP3* and who was successfully treated with anti-IL6 receptor biologic tocilizumab (RoActemra). We discovered a novel heterozygous mutation in *TNFAIP3* c.1906C>T, not previously found in ExAC database. Further analysis shows that this single-nucleotide variant at the terminal residue of *TNFAIP3* exon 7 produces an alternatively spliced mRNA resulting in p.His636fs*Ter*1. Additional genetic analysis of family members shows that this variant does segregate with the inflammatory clinical phenotypes. Subsequent functional test show that NF-κB activation, measured as intracellular phosphorylation of p65 in CD14 + monocytes, was more enhanced in the patient compared with healthy controls (HC) following stimulation with LPS. This was associated with higher production of inflammatory cytokines by the patients PBMC in response to LPS and ATP and enhanced activation of NLRP3 inflammasome complex. Furthermore, increased activation of NLRP3 inflammasome was evident systemically, since we detected higher levels of ASC specks in patients’ sera compared with HC. Finally, we used population genetics data from GnomAD to construct a map of both genetic conservation and most probable disease-causing variants in *TNFAIP3* which might be found in future cases of HA20.

## 1 Background

The protein A20, encoded by *TNFAIP3*, plays a crucial role in the negative regulation of inflammation and immunity (1). With its deubiquitinase enzyme activity, A20 is a critical inhibitor of pro-inflammatory signaling pathways, acting on signaling molecules including inhibitor of nuclear factor kappa B (NF-κB) kinase subunit gamma [IKKγ (NEMO)], TRAF6, and receptor-interacting protein kinase 1 (RIPK1) (2). A20 deubiquitination negatively regulates the NF-κB pathway by hydrolyzing K63-linked polyubiquitin chains which would otherwise allow signaling-complex formation, while its E3 ligase activity has been shown to conjugate substrates including IKKγ with K48-linked ubiquitin chains to target them for proteasomal degradation, and therefore preventing phosphorylation of IκB and translocation of NF-κB (2, 3). A20 haploinsufficiency (HA20) has been recently identified as a cause of early-onset autoinflammatory disease (4). The role of A20 in ubiquitin-dependent disease and otulipenia in NF-κB-dependent autoinflammatory disease has been summarized recently (5).

The clinical phenotype of patients with HA20 varies greatly. The diagnoses in these cases, prior to establishing the genetic cause, included both autoimmune and autoinflammatory disorders and ranged from Behçet’s disease (BD), hereditary fever-like condition, juvenile idiopathic arthritis (JIA) to systemic lupus erythematosus (SLE), and rheumatoid arthritis (RA) (5, 6); however, no patients with HA20 have previously been diagnosed with adult-onset Stills’ disease (AOSD). Here, we present the first patient with HA20, who was previously diagnosed with AOSD but later found to have a novel heterozygous variant in *TNFAIP3* and who was successfully treated with anti-IL6 receptor biologic tocilizumab (RoActemra).

Gene essentiality is measured by quantifying the rate of loss-of-function (LoF) variants from population genetics data. The probability of loss-of-function intolerance (pLi) score for A20 is equal to 1, indicating its intolerance to haploinsufficiency (7). Considering the heterogeneity of HA20 phenotypes and the difficulties in pre-selecting patients for genetic testing, we are likely to find many additional cases of HA20. We used population genetics data from GnomAD to construct a map of both genetic conservation and variant probability. Having a useful guide to predict the presentation of these novel variants and allow for pre-emptive functional investigations, should aid the diagnosis of such cases in the future.

## 2 Case Presentation

A 32-year-old female patient initially presented at the age of 16 with high fever and severe abdominal pain associated with anorexia, but not diarrhea and vomiting. Over the period of the next 4 years, she had multiple hospital admission with similar presentation, which was always accompanied with raised C-reactive protein (CRP). On few occasions her urine dipstick showed traces of protein, and consequently she was treated for presumed urinary sepsis. However, multiple blood and urinary cultures were negative, other investigations including abdominal ultrasound showed no obvious intraabdominal pathology, and the diagnosis remained inconclusive. At the age of 21, she developed sudden onset polyarthritis, fever, and wide spread erythematous skin rash. The routine investigations at the time showed highly elevated acute phase response with CRP > 200 mg/L and hyperferritinemia. Additional investigations including antinuclear antibody (ANA), rheumatoid factor (RF), and anti-citrullinated peptide antibodies (ACPA) were all negative. Diagnosis of AOSD was at that point established, based on the typical clinical features associated with hyperferritinemia and exclusion other autoimmune rheumatological diagnoses. Her condition improved following initial treatment with oral corticosteroids (prednisolone 40 mg daily), but she remained dependant on prednisolone for the disease control despite attempts to introduce disease-modifying antirheumatic drugs (DMARDs) such as hydroxychloroquine, azathioprine, and methotrexate, which were all largely ineffective. Over the next several years, she continued to suffer from inflammatory polyarthritis affecting predominantly the large joints (hips and knees). Prior to establishing her genetic diagnosis, the patient was eventually treated with tocilizumab to which she made an excellent response. She discontinued long-term corticosteroid treatment and remains well controlled on monotherapy with tocilizumab (IV 8 mg/kg monthly) for the last 2 years.

Subsequent enquiries into her family history revealed that she has two children, a boy aged 6 and a half and a girl of age 4 years. Both children had history of unexplained inflammatory symptoms. The girl was symptomatic from the age of 8 months, at which point she begun having recurrent episodes of feeling generally unwell, with malaise and high fever up to 40°C. The frequency of these episodes varied between 2 and 4 weeks, and each episode was relatively short-lived, lasting anywhere between 2 and 3 days. On few occasions, the episodes of fever were associated with erythematous macular rash and mouth ulcers. Subsequently, she also developed episodic abdominal pain as well as difficulty walking due to the pain in her knees. All these symptoms would disappear when the fevers resolved and she would fully recover in between these episodes. Since commencing regular colchicine, she has not had any further inflammatory episodes or symptoms. Her brother was well until age 5 when, like his sister he started with monthly episodes of high fever lasting between several hours to several days. On few occasions, these episodes were associated with vomiting, mouth ulcers, and interestingly, salmon-pink skin rash. He was also commenced on colchicine, which in his case has not been as effective, and he is currently awaiting therapeutic trial of tocilizumab. Finally, the father of the index case also appears to have relevant clinical history. He was diagnosed with early onset RA, but the details of symptoms and investigations leading to his diagnosis are lacking since he is looked after by another hospital and he has minimal contact with his daughter with who he has not shared any further details regarding his health.

## 3 Clinical and Laboratory Investigations

**Figure 1:**
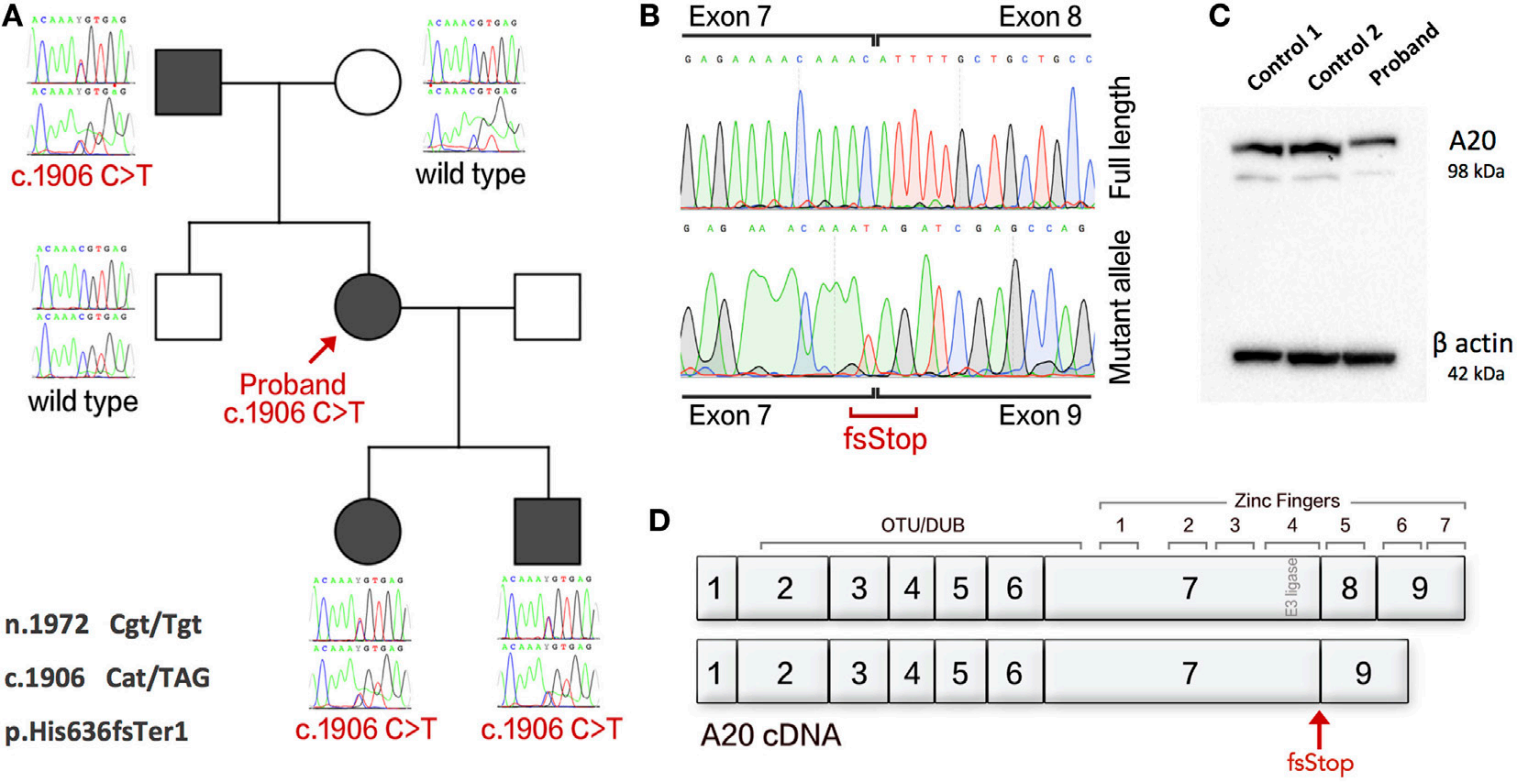
An exonic splice variant in *TNFAIP3* results in deletion of exon 8 in cDNA and A20 haploinsufficiency. **(A)** The Sanger sequences of gDNA in family members showing disease segregation. **(B)** cDNA of proband with separated wild-type and splice variant alleles showing deletion of exon 8. **(C)** Western blot analysis of proband and two healthy controls showing HA20. **(D)** Gene structure of A20 cDNA showing functional domains and the resulting splice variant. Protein purified from PBMCs using sodium orthanovanadate, Complete Protease Inhibitor Cocktail and PMSF (Sigma), with RIPA buffer. Western blotting used an antibody to A20/TNFAIP3 (D13H3) (N-term) Rabbit mAb 5630S (Cell Signaling Technology). The Novex Mini Gel Tank and blot module, Bolt 4–12% Bis–Tris Plus Gels, and PVDF Transfer Membrane (Thermo Fisher Scientific). Imaging used Super Signal West Femto/Pico (Thermo Fisher Scientific).

The patient received genetic testing after purifying proband genomic DNA from whole blood using QIAamp DNA Blood kit (Qiagen). Exome sequencing was performed using Agilent SureSelectXT with All Exon v5 capture library and sequenced on Illumina HiSeq 3000 for 2 × 150-bp paired-end sequencing. A novel heterozygous variant was identified in *TNFAIP3*; ENST00000237289 c.1906C>T (GRCh37 6:138200488 rs200138929). This variant was not found in ExAC database and has allele frequency 2.191e−5 on GnomAD. The proband’s two children also showed a similar phenotype and were also investigated for autosomal dominant inheritance. Genetic analysis of family members found segregation with the inflammatory clinical phenotypes (Figure 1A). PCR clean-up was performed with ExoSAP-IT (Affymetrix). Sanger sequencing using BigDye Terminator Cycle Sequencing Kit, version 3.1 (Applied Biosystems) and analysis on an ABI 3130XL DNA analyzer (Applied Biosystems).

The single-nucleotide variant (SNV) c.1906C>T was first thought to cause a missense mutation. However, further analysis shows that this SNV occurs as the terminal residue of *TNFAIP3* exon 7 (n.1972 Cgt/Tgt) and produces an alternatively spliced mRNA transcript resulting in c.1906 Cat/TAG, p.His636fs*Ter*1. To assess mRNA splicing, we used cDNA synthesized from the proband’s PBMCs and compared with healthy control (HC). The patient’s cDNA produced a mutant allele containing deletion of exon 8 (Figure 1B). Loss of transcript by nonsense-mediated decay resulting in A20 haploinsufficiency was confirmed by Western blot (Figure 1C). The gene structure of A20 is illustrated in Figure 1D. Gel purification of DNA used PureLink™ Quick Gel Extraction Kit (Thermo Fisher Scientific). cDNA was synthesized using the GoScript™ Reverse Transcriptase kit (Promega).

**Figure 2:**
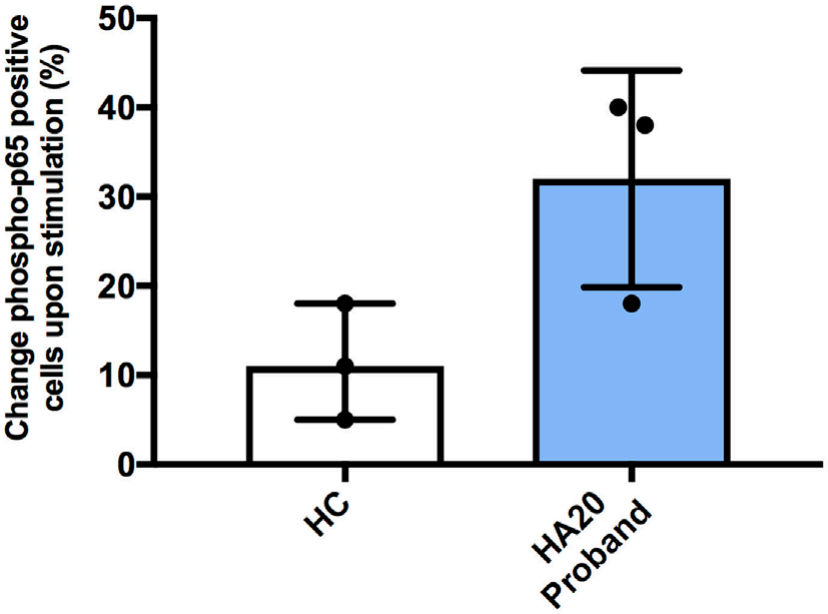
Increased NF-κB phospho-p65 response in A20 haploinsufficiency. The difference in percentage change of cells positive for CD14 and phospho-p65 from unstimulated to stimulated, for healthy controls and A20 proband. Representing three independent experiments. Phosflow with PBMCs were stimulated for 15 min at 37°C with 100 ng/mL LPS (Invivogen, UK). BD Phosflow Fixing and BD Phosflow Perm Buffer III (BD Biosciences) were used in combination with aqua dye Live/Dead Fixable cell stain kit (Invitrogen, UK). Antibodies: CD19 BV421 (Cat No. 562440—clone HIB19); CD3 FITC (Cat No. 555332—clone UCHT1); CD14 AF700 (Cat No. 557923—clone M5E2); CD4 APC-Cy7 (Cat No. 341115—clone SK3/Leu3a); phosphorylated-p65 PE anti-NF-κB pS529 (Cat No. 558423). The BD LSRII 4 laser instrument was used and analyzed with FACS Diva (BD Biosciences).

Previous studies have shown that patients with HA20 show enhanced activation of NF-κB and consequently produce higher levels of various pro-inflammatory cytokines. Therefore, we studied NF-κB activation using flow cytometry (Phosflow system, BD Biosciences) by measuring intracellular phosphorylation of p65 in CD14 + monocytes in response to stimulation with LPS. Following stimulation, greater percentage of patient monocytes became p65 positive compared with monocytes from healthy controls. However, this did not reach a statistical significance (Figure 2). Similarly, spontaneous phosphorylation of p65 NF-κB has been recently reported for a patient with *TNFAIP3* deletion (8).

To determine if this tendency toward enhanced NF-κB activation was associated with increase in cytokine production, the purified PBMC from the patient and HC were treated with a combination of LPS and ATP and various cytokines were measured by ELISA (Thermo Fisher Scientific, IL-1β and IL-18) and by multiplexed particle based flow cytometry [Fluorokinemap (R&D Systems) on a Luminex analyzer (Bio-Plex, Bio-Rad, UK)].

The combination of LPS and ATP was used since we also wanted to investigate activation of NACHT, LRR, and PYD domain-containing protein 3 (NLRP3) inflammasome complex. The NLRP3 inflammasome, which is responsible for processing of pro-IL-1β and pro-IL-18 into their active components, is known to be regulated by A20 (9, 10). Furthermore, in patients with HA20, the NLRP3 inflammasome was previously shown to be constitutively activated (4).

**Figure 3:**
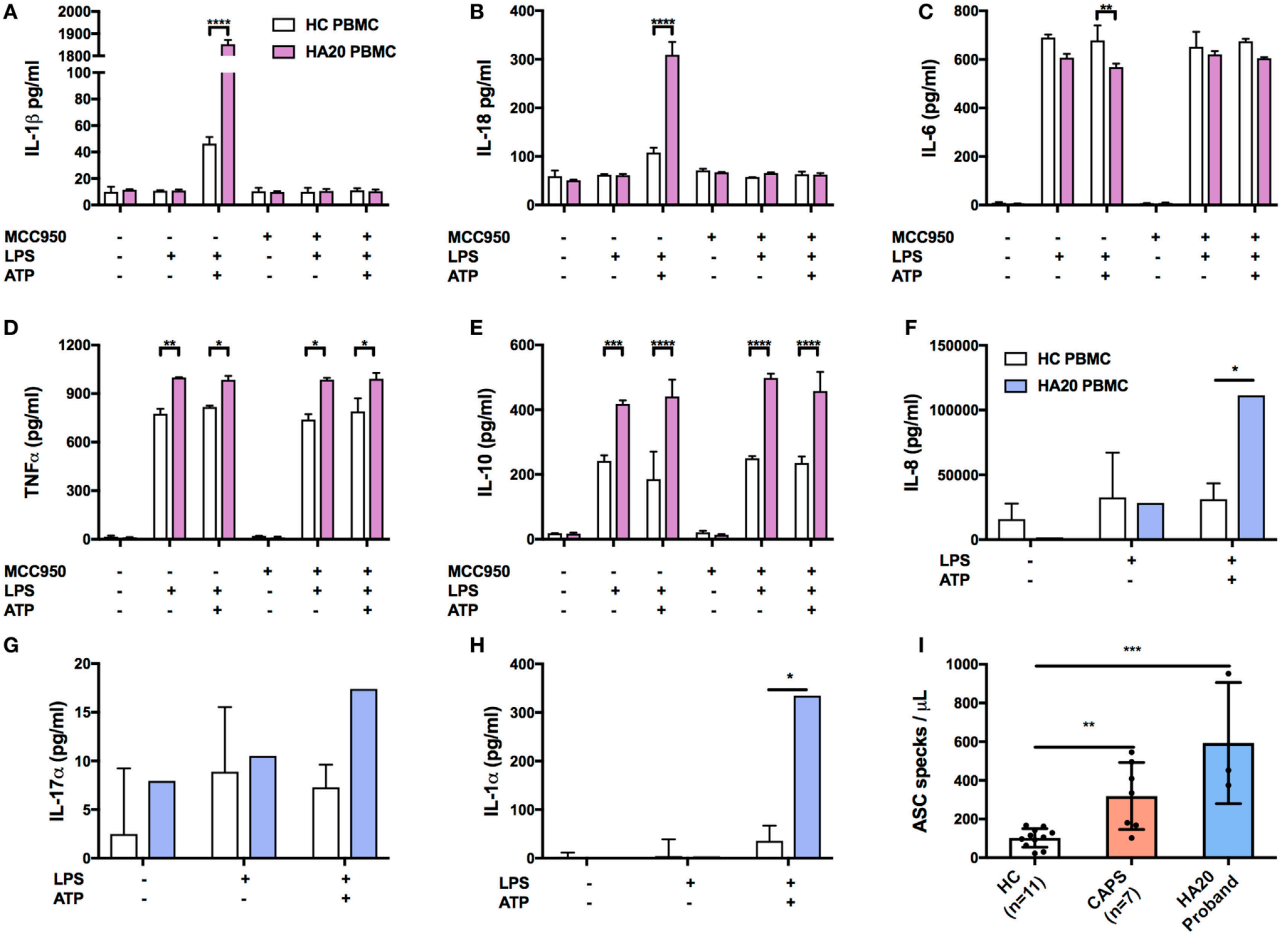
Pro-inflammatory response in A20-deficient patient. Cytokines were measured by ELISA using supernatant from patient and healthy donors. The median confidence interval presented from two separate patient samples. PBMCs were treated with or without MCC950 (NLRP3 inhibitor) and stimulated for 4 h with LPS (10 ng/mL) followed by 1 h ATP (1 mM). Pro-inflammatory responses to NLRP3-dependent activation of **(A)** IL-1β and **(B)** IL-18, and in measurements of **(C)** IL-6, **(D)** TNFα, **(E)** IL-10, **(F)** IL-8 xtbf, **(G)** IL-17α, and **(H)** IL-1α. The median confidence interval or repeated patient measurements is presented. A two-way ANOVA statistical test was performed (*p< 0.05, **p< 0.01, ***p< 0.001, and ****p< 0.0001). **(I)** ASC specks found in the HA20 patient at twice the normal range indicate NLRP3 inflammasome activation and pyroptosis. Treatment concentrations as described in panel **(A)**. Measurements were taken for 7 CAPS patients which also represent a pro-inflammatory response although not as profound as the HA20 response. ASC specs were measured as previously described (11).

The production of IL-1β and IL-18 was found to be significantly increased in the patient’s PBMC compared with healthy controls after stimulation by priming and activation with LPS and ATP, respectively (Figures 3A,B). This effect was attenuated if the cells were pre-treated with the NLRP3 inhibitor, MCC950, thus confirming that IL-1β and IL-18 production was dependant on NLRP3 inflammasome activation. A significant response was also measured in TNFα, and IL-10 but interestingly not in IL-6 (Figures 3C–E). Similarly, the production of pro-inflammatory cytokines IL-17α and IL-1α and chemokine IL-8 were all increased compared with healthy controls (Figures 3F–H).

To determine if enhanced NLRP3 inflammasome activation is also evident systemically, we investigated the patient’s serum and found increased release of apoptosis associated speck-like protein containing a CARD (ASC) specks, which are typically released by cells following activation of the NLRP3 inflammasome and consequent cell death by pyroproptosis (12). Serum (200 mL) was incubated for 1 h with 5 mL of PE anti-ASC (TMS-1) antibody (Cat. 653904, BioLegend, UK). Flow cytometry was performed on a LSRII (BD Biosciences, UK). Non-fluorescent microspheres (1 mm) (Cat. F13838 Thermo Fisher Scientific, UK) were used for gating and ASC specks were quantified in comparison to healthy controls (ASC speck/mL) as previously described (11). We found that patient serum contained significantly higher numbers of ASC specks compared with our healthy control range (Figure 3I).

**Figure 4:**
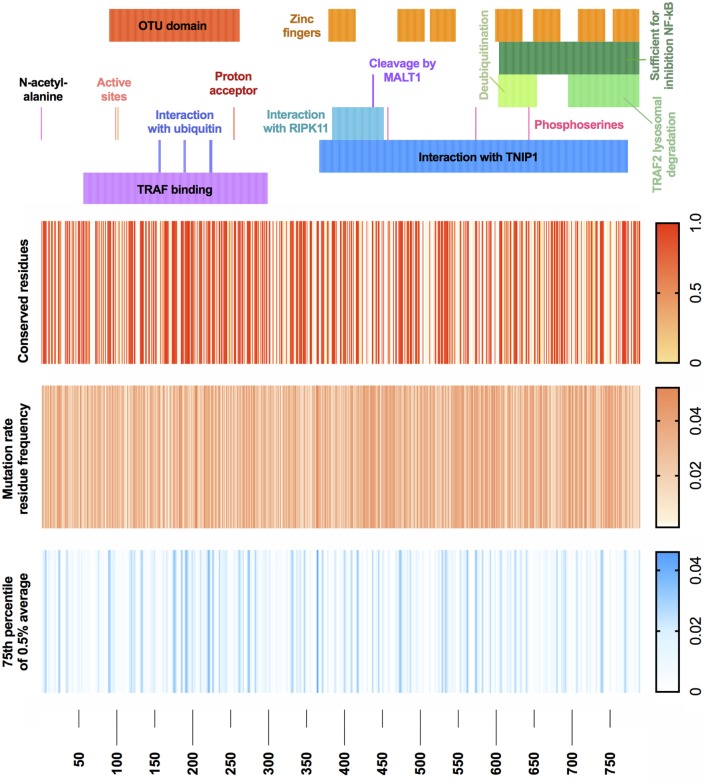
A20 conservation and mutation rate residue frequency. (Top) Gene structure with functional domains. (Heatmap 1) Population genetics conservation rate, non-conserved 0 and conserved 1. (Heatmap 2) Raw MRF score prediction for conserved residues, graded 0 to 0.05. (Heatmap 3) MRF score averaged with 0.5% intervals for each respective gene and cutoff below 75th percentile, graded 0 to 0.045 (noise reduction method). Population genetics data used GnomAD (version r2.0.2); median number of alleles was 245,478 (SD 97,833) covering the *TNFAIP3* canonical transcript ENST00000237289 (GRCh37 6:138188351—138204449). Filtering retained variants with the identifiers: frameshift, inframe deletion, inframe insertion, missense, stop lost, or stop gained. Reference FASTA was sourced from Ensembl TNFAIP3-201 ENST00000237289.8 [HGNC:11896].

With the recent discovery of A20 haploinsuffiency underlining an early-onset autoinflammatory disease (4), relatively few variants are currently identified as disease causing. We wondered what genetic variants are likely to be seen clinically as disease causing in future cases. Population genetics data from GnomAD were used to define the mutation rate residue frequencies of A20 (7). A map of both genetic conservation and most probable disease-causing variants is illustrated in Figure 4 in context with the known functional domains of A20. Raw data and reference values are provided in Table S1 in Supplementary Material. GnomAD was queried to identify conserved residues using a Boolean score C (0 or 1, although allele frequency can be substituted). Regions with no reported variants are highlighted in red (score 1) on the conserved residues heatmap. The gene-specific mutation rate of each residue was calculated from allele frequencies. The gene-specific residue frequency was found. Shown in Table S1 in Supplementary Material, these values together calculate the most probable disease-causing variants which have not yet been identified in patients (13). The mutation rate residue frequency (MRF) heatmap displays the raw calculated likelihood of variation. For visualization, a noise reduction method was also applied where the average MRF per 0.5% interval is displayed with a cutoff threshold at the 75th percentile as shown in Table S1 in Supplementary Material. The resulting MRF heatmap allows visualization of the most probable mutations which correctly identified variants in the ovarian tumor domain as most likely to present clinically as confirmed in a recent extended report by Aeschlimann et al. (14).

## 4 Discussion

Unlike other monogenic autoinflammatory disorders, HA20 seems to be associated with broad clinical phenotypes, ranging from typical autoinflammatory conditions such as JIA to more traditional autoimmune diseases such as SLE. There is one reported case where prior clinical diagnosis could not be established, but the label of undifferentiated complex autoimmune disease was used to describe multitude of clinical signs and complications (15). There is also a single case of 7-month-old Japanese child who presented with typical clinical and laboratory features suggestive of autoimmune lymphoproliferative syndrome, but subsequent genetic testing showed heterozygous mutation in *TNFAIP3* resulting in HA20 diagnosis (16). Another Japanese group has just recently published a report on additional 22 cases with confirmed HA20. Most of the patients reported in the study had BD phenotype, like the original cohort reported by Zhou et al. (4), but they also described individuals presenting with SLE, autoimmune hepatitis, nephritic syndrome, and Hashimoto’s thyroiditis (6). Perhaps this is not entirely surprising since common variants in *TNFAIP3* have also been associated with several autoimmune diseases including SLE, RA, and psoriasis (5). Furthermore, murine studies have demonstrated that the disease phenotype might depend on the predominant cell type that is affected. Homozygous A20 deficiency in mice results in early-onset systemic autoinflammatory disease (17). Cell-specific ablation of murine A20 causes disease features representative of clinical presentation in human (2, 5). Mice that have A20 deficiency in myeloid cells develop polyarthritis, similar to RA in humans and murine intestinal inflammation is seen in enterocyte-specific A20 deficiency (18, 19). Cell-specific A20 deficiency in B cell or dendritic cell also produces tissue-dependent response with the development of autoantibodies (20, 21). It is therefore probable that additional factors, likely both genetic and environmental, play an important role in determining the final disease phenotype in HA20. The heterogeneity of HA20 can be also seen within the same family, as illustrated here, with clinical presentation varying from periodic fever-like illness, AOSD to RA.

There is no standardized treatment for HA20 patients. In general, therapeutic approaches have been guided by the patients’ predominant clinical phenotypes. Most patients respond to corticosteroids; however, there is significant toxicity associated with this therapy since it is often required on a long-term basis. Interestingly, there seems to be a heterogeneous response to colchicine, which is traditionally mainly effective in FMF, and has little or no role in treatment of other hereditary fever syndromes. The efficacy in HA20 is at best partial, but some cases, like the younger sibling we presented here might have a more profound response. More recently, functional cytokine assays which have demonstrated elevated pro-inflammatory cytokine responses (4, 5), therefore supporting a more targeted treatment approach. We have also found increased pro-inflammatory responses in IL-1β, IL-18, TNFα, IL-10, IL-17α, IL-1α, and IL-8, but interestingly not in IL-6. However, at the time of testing, the patient was on tocilizumab, which could have interfered with the functional study. Nevertheless, IL-1β, which is typically upstream of IL-6 and stimulates the secretion of this cytokine, was increased in our patient and therefore there is biological plausibility for using anti-IL6 blockade. Other biological therapies such as anti-TNF and anti-IL-1 have also been effective in suppressing the systemic inflammation in number of patients with HA20 (14). Patients with severe and treatment-refractory disease might be considered for hematopoietic stem cell transplant.

Considering that HA20 has such a heterogeneous clinical presentation, pre-selection of patients for genetic testing, based purely on clinical features, is not likely to be sensitive enough to pick up all relevant cases. Conversely, genetic testing is increasingly performed using gene panels or even whole exome/genome sequencing. Consequently, potentially pathogenic heterzygous variants in *TNFAIP3* might be found in patients who seemingly do not appear to have obvious HA20 phenotype. To complicate matters further, somatic pathogenic mutations in *TNFAIP3* have been identified in lymphomas (22). As we have seen from the murine studies, the phenotypic effects of such mutations might be cell-type dependent. For example, myeloid-restricted mutations in the *NLRP3* gene have now been widely accepted to cause an acquired form of CAPS (23). It is certainly possible to envisage similar situation arising with somatic mutations in *TNFAIP3* restricted to relevant cell types. This challenge requires deep sequencing on a pro-inflammatory gene panel with bioinformatic analysis tailored to somatic variant calling.

Most patients to date have been reported with heterozygous loss-of-function truncating variants. Genetic diagnosis will be difficult in the case of *de novo* mutation. Hypomorphic mutations are an important consideration for A20 deficiency. Highly conserved residues indicate gene regions of essentiality. However, population genetics data from GnomAD show 47.3% conservation across A20; a challenge for pre-emptive functional study. To tackle the void of interpreting novel variants of unknown significance, we applied mutation rate residue frequencies to conserved coding regions and identify the most likely variants to present clinically.

## 5 Conclusion

We identify a case of HA20, which responded positively to anti-IL6 receptor treatment. This report expands on the heterogeneity of HA20 phenotypes. We expect to see reports of disease due to missense mutation and provide a map of most probable variants.

## Ethics Statement

The study was performed in accordance with the Declaration of Helsinki. Human samples were collected with ethical approval from Leeds Ethics Committees, with written informed consent from participants before inclusion in the study. A written informed consent was obtained from the participant for the publication of this case report.

## Author Contributions

SS designed the study, collected clinical data, and was the principal clinician in charge of the patients’ care. DL, RA, and SS wrote the manuscript. DL performed genetic analysis and protein quantification. SP and TS performed whole blood inflammasome-related and cytokine experiments. SP performed ASC speck experiments and flow cytometry experiments. LO performed flow cytometry experiments.

## Conflict of Interest Statement

The authors declare that the research was conducted in the absence of any commercial or financial relationships that could be construed as a potential conflict of interest. The reviewer SV and handling Editor declared their shared affiliation.

## Supplementary Material

Table S1 MRF calculation and reference values. A complete list of calculated mutation rate residue frequencies (MRF) from A20 used for illustration of Figure 4. Population genetics data were acquired from GnomAD (version r2.0.2). Mutation rates and residue frequencies specific to *TNFAIP3* were calculated. A Boolean scoring method was applied for conserved gene regions, scored as 0 and multiple variants per residue scored as 1 (for clarity, the variance frequency is also provided for low allele frequency rare variants). The MRF score predicts the likelihood of mutation for conserved residues. Known functional residues were sourced from UniProt and used for domain color coding in Figure 4. High scoring MRF indicate most probable variants for amino acids which are reported as non-mutated on GnomAD.

## Abbreviations

ACPA, anti-citrullinated peptide antibodies; ANA, antinuclear antibody; AOSD, adult-onset Still’s disease; BD, Behçet’s disease; CRP, C-reactive protein; DMARDs, disease-modifying antirheumatic drugs; HA20, A20 haploinsufficiency; IKKγ, inhibitor of nuclear factor kappa B kinase subunit gamma; JIA, juvenile idiopathic arthritis; LOF, loss-of-function; MRF, mutation rate residue frequency; NF-κB, nuclear factor kappa B; pLi, probability of loss-of-function intolerance; RA, rheumatoid arthritis; RF, rheumatoid factor; RIPK1, receptor-interacting protein kinase 1; SLE, systemic lupus erythematosus.

## References

[1] Das T Chen Z Hendriks RW Kool M A20/tnfaip3 in immune cells controls development of autoinflammation and autoimmunity: lessons from mouse models. Front Immunol (2018) 9:104 10.3389/fimmu.2018.00104 29515565 PMC5826380

[2] Catrysse L Vereecke L Beyaert R van Loo G A20 in inflammation and autoimmunity. Trends Immunol (2014) 35(1):22–31. 10.1016/j.it.2013.10.005 24246475

[3] Vereecke L Beyaert R van Loo G. The ubiquitin-editing enzyme a20 (tnfaip3) is a central regulator of immunopathology. Trends Immunol (2009) 30(8):383–91. 10.1016/j.it.2009.05.007 19643665

[4] Zhou Q Wang H Schwartz DM Stoffels M Park YH Zhang Y Loss-of-function mutations in tnfaip3 leading to a20 haploinsufficiency cause an early-onset autoinflammatory disease. Nat Genet (2016) 48(1):67. 10.1038/ng.3459 26642243 PMC4777523

[5] Aksentijevich I Zhou Q Nf-κb pathway in autoinflammatory diseases: dysregulation of protein modifications by ubiquitin defines a new category of autoinflammatory diseases. Front Immunol (2017) 8:399 10.3389/fimmu.2017.00399 28469620 PMC5395695

[6] Kadowaki T Ohnishi H Kawamoto N Hori T Nishimura K Kobayashi C Haploinsufficiency of a20 causes autoinflammatory and autoimmune disorders. J Allergy Clin Immunol (2018) 141(4):1485–8.e11. 10.1016/j.jaci.2017.10.039 29241730

[7] Lek M Karczewski KJ Minikel EV Samocha KE Banks E Fennell T Analysis of protein-coding genetic variation in 60,706 humans. Nature (2016) 536(7616):285. 10.1038/nature19057 27535533 PMC5018207

[8] Viel S Cheyssac E Pescarmona R Besson L Till M Viremouneix L Large deletion in 6q associated to a20 haploinsufficiency and thoracoabdominal heterotaxy. Ann Rheum Dis (2018). 10.1136/annrheumdis-2018-213300 29678940

[9] Walle LV Van Opdenbosch N Jacques P Fossoul A Verheugen E Vogel P Negative regulation of the nlrp3 inflammasome by a20 protects against arthritis. Nature (2014) 512(7512):69. 10.1038/nature13322 25043000 PMC4126806

[10] Duong BH Onizawa M Oses-Prieto JA Advincula R Burlingame A Malynn BA A20 restricts ubiquitination of pro-interleukin-1β protein complexes and suppresses nlrp3 inflammasome activity. Immunity (2015) 42(1):55–67. 10.1016/j.immuni.2014.12.031 25607459 PMC4302274

[11] Mistry A Scambler T Parry D Wood M Barcenas-Morales G Carter C Glucose-6-phosphatase catalytic subunit 3 (g6pc3) deficiency associated with autoinflammatory complications. Front Immunol (2017) 8:1485. 10.3389/fimmu.2017.01485 29163546 PMC5681747

[12] Galluzzi L Vitale I Aaronson SA Abrams JM Adam D Agostinis P Molecular mechanisms of cell death: recommendations of the nomenclature committee on cell death 2018. Cell Death Differ (2018) 25:486–541. 10.1038/s41418-017-0012-4 29362479 PMC5864239

[13] Lawless D Walter JE Anwar R Savic S Characterising rag1 and rag2 with Predictive Genomics. bioRxiv (2018) 272609 10.1101/272609

[14] Aeschlimann FA Batu ED Canna SW Go E Gül A Hoffmann P A20 haploinsufficiency (ha20): clinical phenotypes and disease course of patients with a newly recognised nf-kb-mediated autoinflammatory disease. Ann Rheum Dis (2018) 77(5):728–35. 10.1136/annrheumdis-2017-212403 29317407

[15] Duncan CJ Dinnigan E Theobald R Grainger A Skelton AJ Hussain R Early-onset autoimmune disease due to a heterozygous loss-of-function mutation in tnfaip3 (a20). Ann Rheum Dis (2018) 77(5):783–6. 10.1136/annrheumdis-2016-210944 28659290 PMC5909743

[16] Takagi M Ogata S Ueno H Yoshida K Yeh T Hoshino A Haploinsufficiency of tnfaip3 (a20) by germline mutation is involved in autoimmune lymphoproliferative syndrome. J Allergy ClinImmunol (2017) 139(6):1914–22. 10.1016/j.jaci.2016.09.038 27845235

[17] Lee EG Boone DL Chai S Libby SL Chien M Lodolce JP Failure to regulate TNF-induced Nf-κB and cell death responses in A20-deficient mice. Science (2000) 289(5488):2350–4. 10.1126/science.289.5488.2350 11009421 PMC3582399

[18] Matmati M Jacques P Maelfait J Verheugen E Kool M Sze M A20 (tnfaip3) deficiency in myeloid cells triggers erosive polyarthritis resembling rheumatoid arthritis. Nat Genet (2011) 43(9):908. 10.1038/ng.874 21841782

[19] Vereecke L Sze M Mc Guire C Rogiers B Chu Y Schmidt-Supprian M Enterocyte-specific a20 deficiency sensitizes to tumor necrosis factor-induced toxicity and experimental colitis. J Exp Med (2010) 207(7):1513–23. 10.1084/jem.20092474 20530205 PMC2901067

[20] Chu Y Vahl JC Kumar D Heger K Bertossi A Wójtowicz E B cells lacking the tumor suppressor tnfaip3/a20 display impaired differentiation and hyperactivation and cause inflammation and autoimmunity in aged mice. Blood (2011) 117(7):2227–36. 10.1182/blood-2010-09-306019 21088135

[21] Hövelmeyer N Reissig S Thi Xuan N Adams-Quack P Lukas D Nikolaev A A20 deficiency in b cells enhances B-cell proliferation and results in the development of autoantibodies. Eur J Immunol (2011) 41(3):595–601. 10.1002/eji.201041313 21341261

[22] Ma A Malynn BA. A20: linking a complex regulator of ubiquitylation to immunity and human disease. Nat Rev Immunol (2012) 12(11):774. 10.1038/nri3313 23059429 PMC3582397

[23] Rowczenio DM Gomes SM Aróstegui JI Mensa-Vilaro A Omoyinmi E Trojer H Late-onset cryopyrin-associated periodic syndromes caused by somatic nlrp3 mosaicism—UK single centre experience. Front Immunol (2017) 8:1410 10.3389/fimmu.2017.01410 29163488 PMC5671490

